# Mild Therapeutic Hypothermia Protects the Brain from Ischemia/Reperfusion Injury through Upregulation of iASPP

**DOI:** 10.14336/AD.2017.0703

**Published:** 2018-06-01

**Authors:** Xiangrong Liu, Shaohong Wen, Shunying Zhao, Feng Yan, Shangfeng Zhao, Di Wu, Xunming Ji

**Affiliations:** ^1^China-America Joint Institute of Neuroscience, Xuanwu Hospital, Capital Medical University, Beijing, China.; ^2^ Beijing Key Laboratory of Translational Medicine for Cerebrovascular Diseases, Beijing, China.; ^3^Cerebrovascular Diseases Research Institute, Xuanwu Hospital of Capital Medical University, Beijing, China.; ^4^Department of Neurosurgery, Beijing Tongren Hospital, Capital University of Medical Sciences, Beijing, China.; ^5^Department of Neurosurgery, Xuanwu Hospital of Capital Medical University, Beijing, China.

**Keywords:** apoptosis, iASPP protein, stroke, hypothermia, therapeutic, small interfering RNA

## Abstract

Mild therapeutic hypothermia, a robust neuroprotectant, reduces neuronal apoptosis, but the precise mechanism is not well understood. Our previous study showed that a novel inhibitor of an apoptosis-stimulating protein of p53 (iASPP) might be involved in neuronal death after stroke. The aim of this study was to confirm the role of iASPP after stroke treated with mild therapeutic hypothermia. To address this, we mimicked ischemia/reperfusion injury in vitro by using oxygen-glucose deprivation/reperfusion (OGD/R) in primary rat neurons. In our in vivo approach, we induced middle cerebral artery occlusion (MCAO) for 60 min in C57/B6 mice. From the beginning of ischemia, focal mild hypothermia was applied for two hours. To evaluate the role of iASPP, small interfering RNA (siRNA) was injected intracerebroventricularly. Our results showed that mild therapeutic hypothermia increased the expression of iASPP and decreased the expression of its targets, Puma and Bax, and an apoptosis marker, cleaved caspase-3, in primary neurons under OGD/R. Increased iASPP expression and decreased ASPP1/2 expression were observed under hypothermia treatment in MCAO mice. iASPP siRNA (iASPPi) or hypothermia plus iASPPi application increased infarct volume, apoptosis and aggravated the neurological deficits in MCAO mice. Furthermore, iASPPi downregulated iASPP expression, and upregulated the expression of proapoptotic effectors, Puma, Bax and cleaved caspase-3, in mice after stroke treated with mild therapeutic hypothermia. In conclusion, mild therapeutic hypothermia protects against ischemia/reperfusion brain injury in mice by upregulating iASPP and thus attenuating apoptosis. iASPP may be a potential target in the therapy of stroke.

Although ischemic stroke is one of the major causes of death and disability worldwide, hitherto, tissue plasminogen activator is the only effective drug for ischemic stroke patients but has a relatively narrow therapeutic time window of 4.5 h [1]. Only 1%-7% of stroke patients receive tissue plasminogen activator treatment [1]. Mild therapeutic hypothermia (32°C-35°C) has been recognized as an effective neuroprotectant in experimental stroke models as well as patients with stroke and extends the therapeutic window for other neuroprotectants [2-6]. Multiple mechanisms, such as inhibition of apoptosis, necrosis and inflammatory response, are involved in neuroprotection of mild therapeutic hypothermia after cerebral ischemia [5-7]. A clearer understanding of the changes in signaling pathways and their downstream targets is critical to the development of therapeutic interventions to alleviate cerebral ischemia/reperfusion injury.

The *p53* gene family consists of *p53* and its related genes, *p63* and *p73* [8]. As reported previously, the transcriptional activator p53 is a key regulator of neuronal cell death after cerebral ischemia injury [9, 10]. Inhibition of the function of p53 can reduce apoptosis and induce endogenous nerve regeneration in ischemic brain injury [11, 12]. The role of p53 has been discussed in numerous studies, while the effect of p63 and p73 in cerebral ischemia are still rarely investigated. There are currently three known members of the apoptosis-stimulating proteins of the p53 (ASPP) family, ASPP1, ASPP2 and iASPP, an inhibitory member of ASPPs [13]. These family members bind p53 family proteins (p53, p63, and p73) via a high degree of homology at their carboxyl terminus [13, 14]. ASPP1 and ASPP2 facilitate the apoptotic function of the p53 family [13-15]. Due to different components in the amino terminus, iASPP competes with ASPP1 and ASPP2, and thereby inhibits p53-dependent and -independent apoptosis [13-15]. A previous study showed that iASPP is required for neuronal survival after axonal injury [16]. We also found that the ASPP family may be involved in neuronal cell death after stroke [17]. Moreover, ischemic stroke was associated with upregulation of ASPP1 and ASPP2 and downregulation of iASPP [17]. These data indicate that iASPP may play an important role after stroke.

The present study aims to gain new insight into the role of iASPP after experimental stroke and the effect of iASPP on hypothermia-induced neuroprotection. Here, we use oxygen-glucose deprivation/reperfusion (OGD/R) and a middle cerebral artery occlusion (MCAO) mice model to mimic ischemia/reperfusion injury *in vitro* and *in vivo* to detect the role of iASPP in cerebral ischemia treated with local mild therapeutic hypothermia. Our results demonstrated that mild hypothermia upregulated iASPP and downregulated its targets related to apoptosis. Downregulation of iASPP by small interfering RNA (siRNA) aggravated focal cerebral ischemia injury in MCAO mice treated with mild therapeutic hypothermia or normothermia, proposing iASPP as a potential therapeutic target for ischemic stroke.

## MATERIALS AND METHODS

### Primary culture of cortical neurons

Primary neurons were cultured as previously described [18]. Briefly, primary neurons were obtained from cerebral cortices of 18-day-old Sprague-Dawley rat embryos, and cultured in Neurobasal Medium (Gibco, Life Tech, Gaithersburg, MD, USA) supplemented with 2% B27 (Life Tech, Grand Island, NY, USA) and 1% GlutaMAX (Life Tech). The cells were incubated at 37°C in a humidified atmosphere of 95% O_2_/5% CO_2_. The medium was replaced after a day of incubation and neurons were cultured for additional nine days before use.

### OGD/R

Ten days old cortical neuron cultures were exposed to OGD/R. Briefly, the cultured medium was replaced by glucose-free Dulbecco’s Modified Eagle Medium (Life Tech), and placed in a hypoxic chamber (Billups Rothenberg, Inc., Del Mar, CA, USA) containing 5% CO_2_, 0.02% O_2_ and 94.98% N_2_ at 37°C. After an hour of OGD, the glucose-free Dulbecco’s Modified Eagle Medium was displaced by the original culture media and neurons were returned to normal cultured conditions for 24 h.

### Western blot analysis

Cerebral tissues were sampled at 4, 12, 24 and 72 h after ischemia whereas cultured cells were collected at 24 h after OGD. Western blots were performed using protocols as previously described [17]. The following antibodies were used: rabbit anti-ASPP1 polyclonal antibody (1:200, Abbiotec, San Diego, CA, USA), rabbit anti-ASPP2 polyclonal antibody (1:200, Abbiotec), rabbit anti-iASPP polyclonal antibody (1:1000, Abcam, Hong Kong, China), rabbit anti-p53-upregulated mediator of apoptosis (Puma) (1:1000, Abcam), rabbit anti-Bax (1:200, Santa Cruz Biotechnology Inc., Santa Cruz, CA, USA), cleaved caspase-3 (1:1000, Cell Signaling Technology Inc. Beverly, MA, USA) and rabbit anti-Actin polyclonal antibody (1:2000, Santa Cruz Biotechnology Inc.). Membranes were then incubated with peroxidase-conjugated goat anti-rabbit immunoglobulin G (1:2000, Santa Cruz Biotechnology Inc.). The blots were visualized by chemiluminescence (Millipore, Billerica, MA, USA). Protein levels were normalized to β-Actin as a loading control. The relative optical density of protein bands was measured after subtracting the film background.

### Immunofluorescence staining

Immunofluorescence staining was performed on coverslips in 24-well plates or 25 μm brain sections. Primary antibodies included rabbit polyclonal anti-iASPP antibody (diluted 1:100; Abcam), mouse monoclonal anti-microtubule-associated protein 2 (MAP2; 1:50; Santa Cruz Biotechnology Inc.), rabbit polyclonal anti-ASPP1 antibody (1:100; Abbiotec), rabbit polyclonal anti-ASPP2 antibody (1:100; Abbiotec) and mouse monoclonal anti-NeuN antibody (1:100; Millipore, Billerica, MA, USA). Goat anti-rabbit secondary antibody (tetramethylrhodamine-conjugated) (1:400; Jackson Immuno Research Laboratories Inc, West Grove, PA, USA) and goat anti-mouse secondary antibody (fluorescein isothiocyanate-conjugated) (1:400; Jackson Immuno Research Laboratories Inc.) were used. The nuclei of cells were stained by 4′6-diamidino-2-phenylindole (DAPI) before taking the images. Cultured neurons were observed in a confocal microscope (Leica, Wetzlar, Germany) whereas tissue sections were observed using a ?uorescence microscope (Carl Zeiss, Jena, Germany).

### Animal model

All animal experiments were approved by the Institutional Animal Care and Use Committee of Capital Medical University and performed as per the principles outlined in the NIH Guide for the Care and Use of Laboratory Animals. Male 10-week-old C57/B6 mice weighing 23-25g were subjected to MCAO for an hour followed by reperfusion as previously described [19]. Right MCAO was performed by inserting a 7-0 nylon monofilament (Doccol Corporation, Sharon, MA, USA) into the internal carotid artery. Body temperature was measured by a thermocouple probe placed in the rectum and was maintained at normothermia (37°C) throughout the experiment by a temperature-regulated heating pad and a heating lamp. The cerebral temperature was measured through a thermocouple probe placed close to the skull under the right temporalis. Small ice bags were placed beneath and around the head and neck areas of the mouse and were replaced every hour. Mild therapeutic hypothermia (33 ± 0.5°C) was applied from the beginning of ischemia for a duration of 2 h. Infarct volume and neurological outcomes at 24 h after ischemia were evaluated. The mice were randomly assigned into eight groups: sham-operated + 37°C group (n=8), sham-operated + 33°C group (n=8), MCAO + 37°C group (n=31), MCAO + 33°C group (n=32), MCAO + 37°C + control siRNA (Coni) group (Coni was intracerebroventricularly injected) (n=21), MCAO + 37°C + iASPP siRNA (iASPPi) group (iASPPi was intracerebroventricularly injected) (n=24), MCAO + 33°C + Coni group (n=20), and MCAO + 33°C + iASPPi group (n=21). To ensure the occurrence of ischemia and reperfusion in MCAO animals, regional cerebral blood flow was monitored using laser Doppler flowmetry (PeriFlux System 5000, Perimed, Stockholm, Sweden).

### Neurological function evaluation

Animals were examined at 24 h after cerebral ischemia using a modified neurological severity score (mNSS) as previously described [20-22]. Scores ranged from 0 in healthy animals to a maximum 10 in stroke animals that failed in all tasks. The mNSS contains motor tests and sensory tests. Motor tests were assessed via holding mouse by the tail to detect the flexion of limbs and the angle of the head (normal: 0; maximum: 3) and placing the animal on the floor to detect the way of a walk (normal: 0; maximum: 3). Sensory tests included tactile response and proprioceptive tests. The tactile response was evaluated by touching palmar area of forepaw with a sharp needle (normal: 0; maximum: 2). A proprioceptive test was assessed by pushing mouse from the side of the neck with a cotton swab (normal: 0; maximum: 2). Neurological function was assessed blindly.

### Intracerebroventricular injection

Before MCAO, mice were randomly divided into iASPP siRNA (Santa Cruz) and control siRNA (Santa Cruz) groups. Adult male CB57/B6 mice were anesthetized with 2% isoflurane in 70% N_2_O balanced O_2_ by the facemask and placed in a stereotaxic frame (World Precision Instruments, Sarasota, FL, USA). Intracere-broventricular injection was performed as previously described [23]. iASPP siRNA or control miRNA mixture (siRNA 4.2 μl, lipofectamine 2000 1.2 μl, and ddH_2_O 0.6 μl) were immediately stereotaxically delivered into the ipsilateral lateral ventricle over 10 min. The needle was retained for 5 min after injection. The bone wound was closed with bone wax.

### Infarct volume analysis

Infarct volume was determined using 2, 3, 5-triphenyltetrazolium chloride (Sigma, St. Louis, MO, USA) as previously described [22].

### Terminal transferase-mediated dUTP nick end labeling (TUNEL) staining

TUNEL staining was performed using an *in situ* cell death detection kit according to the manufacturer’s instructions (Roche Applied Science, South San Francisco, CA, USA). The nuclei of cells were stained with DAPI. Sections were observed and measured as previously described [24].

### Statistics

GraphPad Prism 7 software (GraphPad Software Inc., San Diego, CA, USA) was used for all statistical analyses. One-way analysis of variance (ANOVA) followed by Tukey’s test or two-way ANOVA followed by Sidak’s test were used to compare multiple groups. All data are presented as means ± standard errors of the means (SEM). A value of *p* < 0.05 was considered statistically significant.

## RESULTS

### Mild therapeutic hypothermia regulates the expression of iASPP and its targets in primary neurons under OGD/R

**Figure 1.**
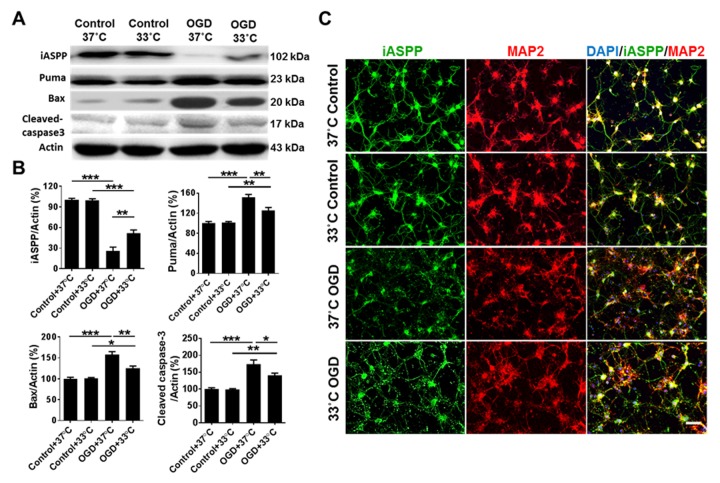
Mild therapeutic hypothermia regulates the expression of iASPP and its targets in primary neurons treated with OGD/R. (A) Representative protein in bands of iASPP, Puma, Bax and cleaved caspase-3 at 24 h after OGD from the Western blot. Actin served as a loading control. (B) Densitometric quantification of iASPP, Puma, Bax and cleaved caspase-3 from the Western blot, n = 4 per group. (C) Representative images showing expression of iASPP (green) in primary neurons (red) treated with OGD at 37°C or 33°C. DAPI was used as a nuclear marker. Bar, 50 μm.*p < 0.05, **p < 0.01, ***p < 0.001, by one-way ANOVA and Tukey’s test.

To detect whether hypothermia affects the expression of iASPP and its targets related to apoptosis *in vitro*, primary cortical neurons were treated with OGD/R and mild therapeutic hypothermia (33°C) or normothermia (37°C). As shown in Fig. 1A and B, the protein level of iASPP was higher (51.78 ± 4.68% in OGD + 33°C group versus 25.96 ± 5.57% in OGD + 37°C group, *p* = 0.003), while the expression of iASPP targets Puma and Bax, and an apoptosis marker, cleaved caspase-3 [25], were lower in OGD-subjected neurons cultured at 33°C than at 37°C (Puma/β-Actin: 125.79 ± 5.56% in OGD + 33°C group versus 151.86 ± 5.53% in OGD + 37°C group, *p* = 0.006; Bax/β-Actin: 125.47 ± 5.09% in OGD + 33°C group versus 157.89 ± 7.11% in OGD + 37°C group, *p* = 0.002; cleaved caspase-3/β-Actin: 141.37 ± 5.92% in OGD + 33°C group versus 174.19 ± 12.15% in OGD + 37°C group, *p* = 0.030). Consistent with the Western blot results, immunostainings showed that the expression of iASPP was particularly induced in OGD-subjected neurons treated with the 33°C culture (Fig. 1C). The expression of MAP2, a marker of neurons which could evaluate neuronal survival [26], was also increased in OGD neurons treated with the 33°C culture (Fig. 1C).

### Mild therapeutic hypothermia increased iASPP expression in MCAO mice

**Figure 2.**
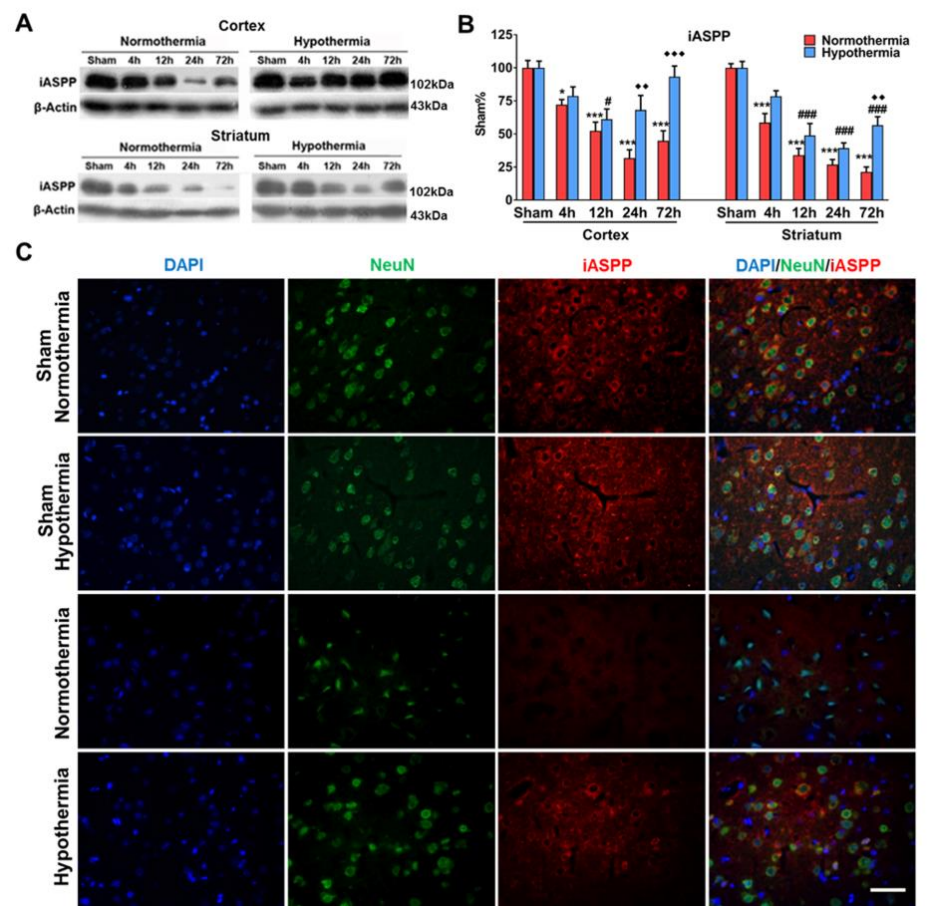
Mild therapeutic hypothermia augmented expression of iASPP after stroke. (A) Representative protein bands of iASPP from the Western blot. Actin served as a loading control. Mice subjected to normothermic or mild hypothermic stroke were euthanized at 4, 12, 24 and 72 h later. (B) Densitometric quantification of iASPP from the Western blot. *p < 0.05, ***p < 0.001, vs sham group under normothermia; # *p* < 0.05, ### *p* < 0.001, vs sham group under mild therapeutic hypothermia; ♦♦ *p* < 0.01, ♦♦♦ *p* < 0.01, vs corresponding groups under normothermia, by two-way ANOVA and Sidak’s test, n = 4 per group. (C) Representative images showing localization of iASPP (red) in neurons (green) of the cortex at 24 h after stroke. Bar, 50 μm.

To evaluate the expression level of iASPP, Western blot and immunostaining were performed on brain samples from sham-operated groups and MCAO mice treated with mild therapeutic hypothermia (33°C) or normothermia (37°C). Normothermia decreased iASPP expression starting from 4 h (72.18 ± 3.81%), followed by a continued decrease at 12 h (52.36 ± 6.60%) and 24 h (31.73 ± 6.31%) and a recovery beginning at 72 h (44.86 ± 7.51%) in the ipsilateral cortex (versus 100.00 ± 5.50% in sham-operated group, *p* = 0.019, *p* < 0.001, *p* < 0.001 and *p* < 0.001, respectively; Fig. 2A and B). Meanwhile, iASPP expression showed a continuous decline in the ipsilateral striatum of MCAO mice under normothermia (all *p* < 0.001; Fig. 2A and B). In comparison with samples from MCAO mice treated with normothermia, the expression level of iASPP significantly increased at 24 h (68.12 ± 10.98% in 33°C group versus 31.73 ± 6.31% in 37°C group, *p* = 0.001; Fig. 2A and B) and 72 h (93.25 ± 8.09% in 33°C group versus 44.86 ± 7.51% in 37°C group, *p* < 0.001; Fig. 2A and B;) after ischemia in the cortex and at 72 h after ischemia in the striatum under the application of mild therapeutic hypothermia (56.59 ± 6.46% in 33°C group versus 21.29 ± 3.78% in 37°C group, *p* = 0.002; Fig. 2A and B). Consistent with the Western blot results, immunostainings showed increased iASPP expression at 24 h after ischemia in the ipsilateral cortex in mild therapeutic hypothermia-treated MCAO mice (Fig. 2C).

### Mild therapeutic hypothermia decreased expression of the family members of iASPP in MCAO mice

**Figure 3.**
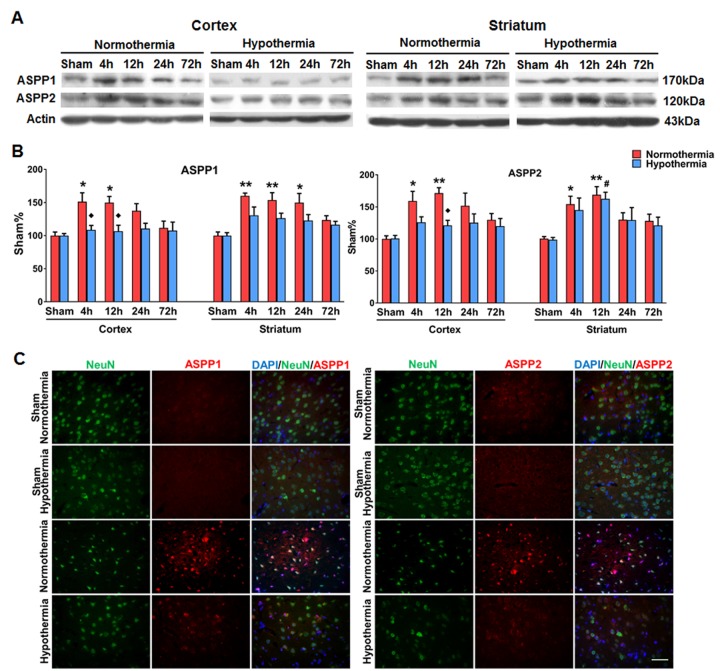
Mild therapeutic hypothermia decreased expression of ASPP1 and ASPP2 after stroke. (A) Representative protein bands of ASPP1 and ASPP2 from the Western blot. Actin served as a loading control. Mice subjected to normothermic or mild hypothermic stroke were euthanized at 4, 12, 24 and 72 h later. (B) Densitometric quantification of ASPP1 and ASPP2 in Western blot, n = 4 per group. Data are expressed as mean ± SEM. *p < 0.05, **p < 0.01, vs sham group under normothermia; # *p* < 0.05 vs sham group under mild therapeutic hypothermia; ♦ *p* < 0.05, vs. corresponding group under normothermia, by two-way ANOVA and Sidak’s test. (C) Representative images showing expression of ASPP1 and ASPP2 (red) in neurons (green) of the cortex at 12 h after cerebral ischemia. DAPI was used as a nuclear marker.

To detect hypothermia-induced expression changes of the proteins that interact with iASPP in p53-independent apoptosis, expression levels of the family members of iASPP were evaluated by Western blot and immunostaining. As shown in Fig. 3A and B, the protein levels of ASPP1 and ASPP2 in the ipsilateral cortex were increased at 4 h and 12 h after ischemia under normothermia. The protein concentration of ASPP1 was lower at 4 and 12 hours and ASPP2 was lower at 12 hours after ischemia in the cortex of hypothermia-treated group compared with the normothermia-treated group (*p* = 0.015, *p* = 0.043 and *p* = 0.013 respectively; Fig. 3A and B). Consistent with the Western blot results, immunostainings also showed that the expression of ASPP1 and ASPP2 was decreased in the ipsilateral cortex of the hypothermia group (Fig. 3C).

### Downregulation of iASPP expression increased infarct volume, aggravated functional outcomes and induced cell death in cerebral ischemia of mice treated with mild therapeutic hypothermia

**Figure 4.**
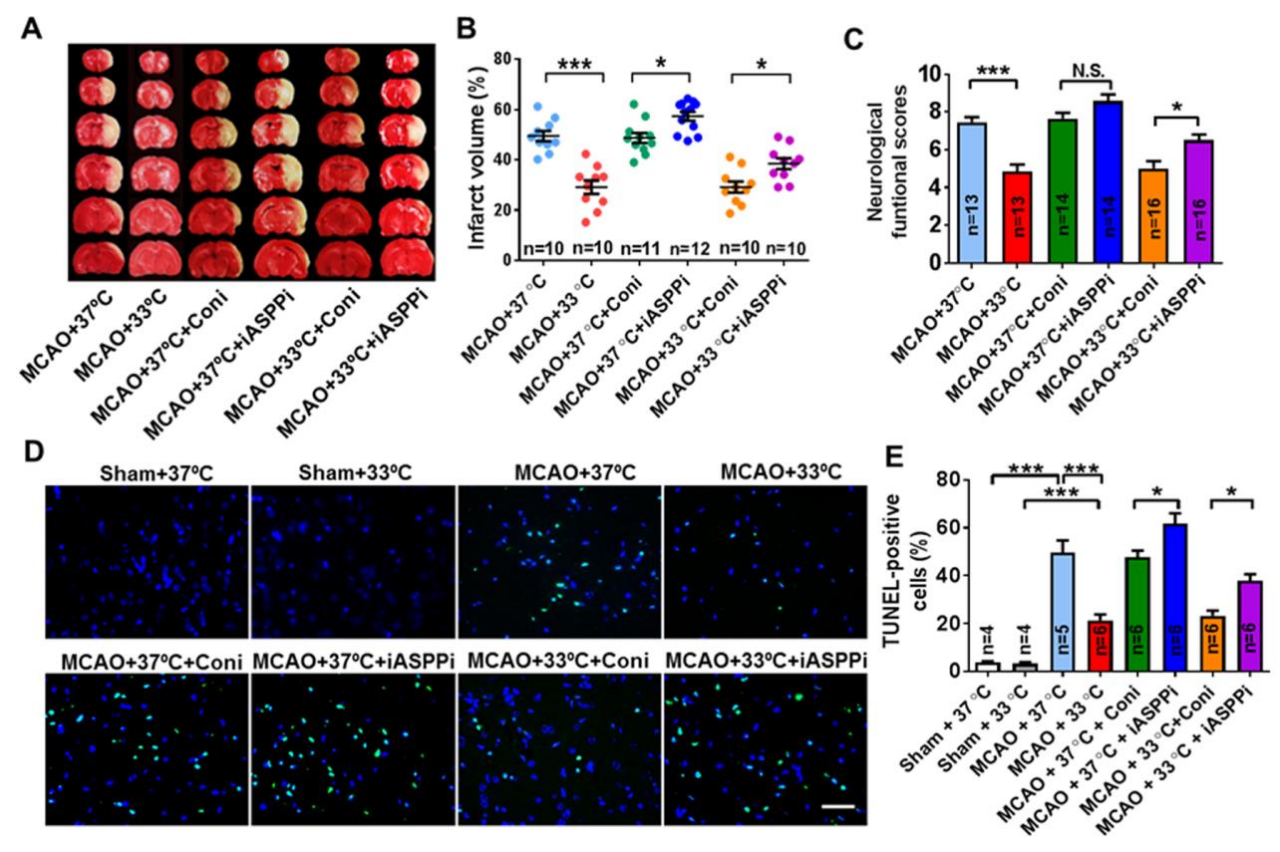
Effect of iASPPi and iASPPi plus hypothermia on mice cerebral ischemic injury. (A) Representative brain slices with infarcts stained by 2, 3, 5-triphenyltetrazolium chloride from each group at 24 h after reperfusion. (B) Statistical analysis of the percentage of infarct volume in different treated groups, n = 10-12 per group. (C) Statistical analysis of neurological severity scores (NSS) in different treated groups, n = 13-16 per group. (D) Representative TUNEL staining (green) of brain slices from different groups at 24 h after reperfusion. DAPI was used as a nuclear marker. (E) Statistical analysis of apoptosis cells showing that mild hypothermia decreased cell apoptosis, n = 4-6 per group. 37°C, normothermia. 33°C, mild therapeutic hypothermia. Small interfering RNA (siRNA) was intracerebroventricularly injected. Coni, control siRNA; iASPP siRNA (iASPPi). Data are expressed as mean ± SEM. *p < 0.05, **p < 0.01, ***p < 0.001, by one-way ANOVA and Tukey’s test.

To further explore the effect of iASPP on stroke treatment with mild therapeutic hypothermia, iASPPi or Coni were injected into the ipsilateral ventricle of mice at 30 min before the beginning of ischemia. The outcome was evaluated 24 h after reperfusion. As shown in Fig. 4A and B, infarct volume and cell death in the MCAO + 37°C + iASPPi group (infarct volume: 57.36 ± 1.77%; cell death: 61.62 ± 4.44%) was increased significantly compared with the MCAO+ 37°C + Coni group (infarct volume: 48.75 ± 1.98%; cell death: 47.75 ± 2.67%) (*p* = 0.046 and *p* = 0.047, respectively). iASPPi application aggravated the neurological function, although not statistically significantly compared with Coni at normothermia (*p* = 0.418; Fig. 4C). iASPPi plus hypothermia increased infarct volume (38.51 ± 2.17% in MCAO + 33°C + iASPPi group versus 29.19 ± 2.23% in MCAO + 33°C + Coni group, *p* = 0.042; Fig. 4A and B), aggravated neurological function (6.5 ± 0.32 in MCAO + 33°C + iASPPi group versus 5.00 ± 0.40 in MCAO + 33°C + Coni group, *p* = 0.020; Fig. 4C) and induced cell death (37.82 ± 2.83 in MCAO + 33°C + iASPPi group versus 23.25 ± 2.21 in MCAO + 33°C + Coni group, *p* = 0.032; Fig. 4D and E), compared with the corresponding Coni group treated with hypothermia. These data suggested that iASPP may have a protective role on the outcome of stroke and stroke treated with mild therapeutic hypothermia.

### Downregulation of iASPP increased the expression of its downstream targets and an apoptosis marker in cerebral ischemia of mice treated with mild therapeutic hypothermia

**Figure 5.**
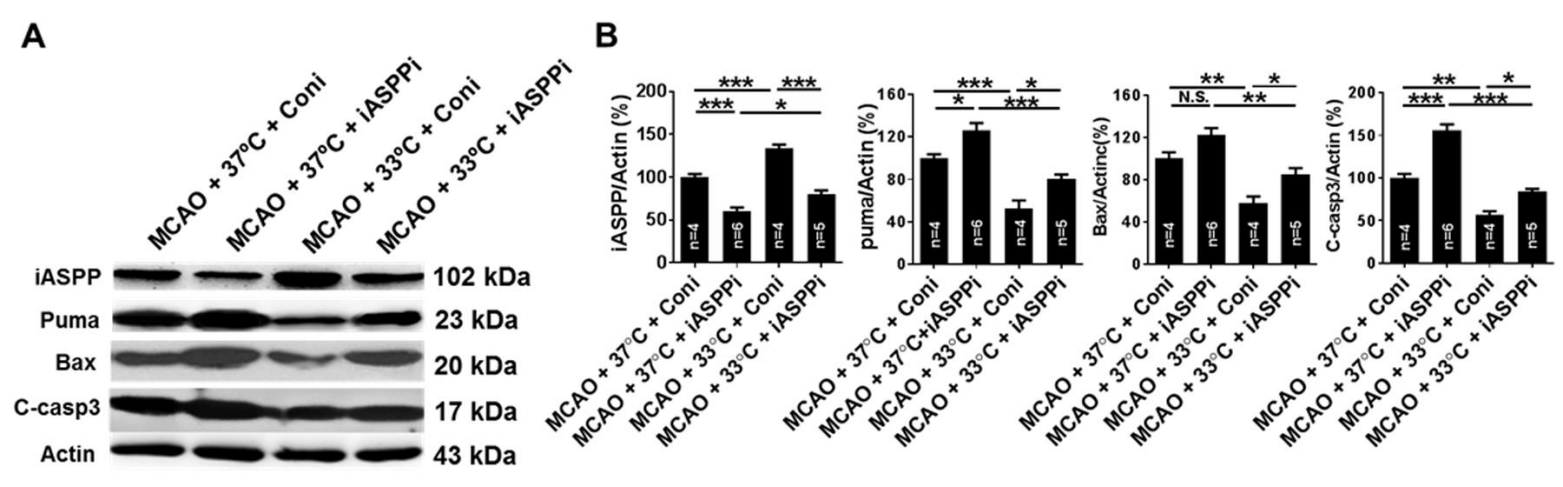
Effect of iASPPi and iASPPi plus hypothermia on the expression of iASPP and its targets in MCAO mice. (A) Representative protein in bands of iASPP, Puma, Bax and cleaved caspase-3 at 24 h after cerebral ischemia from the Western blot. Actin served as a loading control. (B) Densitometric quantification of iASPP, Puma, Bax and cleaved caspase-3 measured by Western blot. C-casp3, cleaved caspase-3, n = 4-6 per group. 37°C, normothermia. 33°C, mild therapeutic hypothermia. Small interfering RNA (siRNA) was intracerebroventricularly injected. Coni, control siRNA; iASPP siRNA (iASPPi). Data are expressed as mean ± SEM. N.S., not significant. *p < 0.05, **p < 0.01, ***p < 0.001, by one-way ANOVA and Tukey’s test.

To further explore the effect of iASPP in cerebral ischemia and therapeutic hypothermia-mediated neuroprotection, ipsilateral hemispheres were obtained at 24 h after ischemia to detect the expression of iASPP and its targets by Western blot. Compared to the MCAO control treated with normothermia (100.00 ± 3.70%), iASPP siRNA resulted in a decreased expression of iASPP (60.18 ± 4.37%, *p* < 0.001; Fig. 5) and an increased expression of Puma and cleaved caspase-3 (*p* = 0.029 and *p* < 0.001, respectively; Fig. 5). The expression of its downstream targets Bax was increased in the iASPPi + normothermia group, although not statistically significantly compared with the Coni group treated with normothermia (*p* = 0.081, Fig. 5). Treatment with siRNA for iASPP grossly reduced iASPP expression (80.80 ± 3.82% in MCAO + 33°C + iASPPi group versus 133.52 ± 4.52% in MCAO + 33°C + Coni group, *p* < 0.001; Fig. 5) and increased the expression of Bax, Puma and cleaved caspase-3 in the MCAO + 33°C + iASPPi group compared with those treated with Coni under hypothermia (*p* = 0.024, *p* = 0.041 and *p* = 0.024, respectively; Fig. 5). The data suggest that iASPPi could increase apoptosis in cerebral ischemia of mice under normothermia or hypothermia treatment.

## DISCUSSION

To our knowledge, this is the first study to describe the beneficial effect of iASPP after stroke, especially if applied with mild hypothermia. In this study, we demonstrated several findings relevant to therapeutic hypothermia. First, mild hypothermia upregulated the expression of iASPP and downregulated its targets in primary neurons under OGD/R. Second, downregulation of iASPP after stroke or stroke plus therapeutic hypothermia increased infarct volume and cell death, and aggravated neurological deficits in MCAO mice. Third, downregulation of iASPP increased the expression of its targets and apoptosis in mice after stroke.

Application of mild hypothermia (32°C-35°C) provides neuroprotection while avoiding serious adverse effects of hypothermia [3, 4, 6]. Hence, 33°C was chosen as the therapeutic temperature in this study. Therapeutic hypothermia has been extensively studied as one of the most robust neuroprotectants against neuronal apoptosis and survival in cerebral ischemia in the laboratory and clinic, which affects multiple pathological pathways after stroke [7].

Consistent with the collective findings of previous studies about the effects of hypothermia on neuronal survival following cerebral ischemia/reperfusion injury [7, 27], our data showed that therapeutic hypothermia improved neurological function and decreased apoptosis, accompanied by increased expression of iASPP. As a family member of ASPPs, iASPP was initially found and studied in cancer [28], which is associated with dysregulated cellular processes, such as cell cycle alterations and apoptosis [29, 30]. iASPP has often been shown to be upregulated in numerous types of human malignancy, such as leukemia [31], glioma [32] and cervical adenocarcinoma [33]. Moreover, iASPP enhances self-renewal of hematopoietic stem cells [34]. These studies suggested that iASPP promotes tumor growth in certain types of cancer and may facilitate cell survival and cell growth. A recent study reported that iASPP expression was decreased in cerebral ischemia and was modulated by miR-182 [35], an apoptosis inducer [36], which was similar to our previous study [17] and the present study. Caspase-3 can be activated and cleaved to cleaved caspase-3 as a classical apoptotic marker [25]. Moreover, caspase-3 activation contributes to iASPP cleavage, which inhibits p53 activity [37]. Here, we found that downregulation of iASPP enhanced cell death and increased cleaved caspase-3 expression, suggesting that iASPP may have an anti-apoptotic function after stroke.

As reported, therapeutic hypothermia plays a neuroprotective role by inhibiting apoptosis [5]. iASPP depletion is reported to trigger p53-dependent [38] and p63/p73-dependent apoptosis [14]. P53-dependent apoptosis is an important pathway in the induction of apoptosis [39]. Hypothermia was found to reduce ischemic neuronal death by downregulation of p53 [40]. However, hypothermia may enhance the expression of p53 and its response gene p21 to promote repair after stroke [41], suggesting that p53 may have a dual role after stroke. As members of p53, p63 and p73 are presumed to have a similar function in apoptosis, but p63 and p73 may, in fact, be survival factors rather than death-promoting factors. A previous study showed increasing expression of p63 and p73 in the early stage of cerebral ischemia in rat pups [42]. Ischemic preconditioning protected the reduction of p63 immunoreactivity in gerbil hippocampal neurons following ischemia. P73 was essential for neuronal survival and loss of p73 increased neuronal apoptosis [43, 44]. P73 elicits apoptosis by modulating Puma and Bax [45]. In short, further investigation is needed to elucidate the role of the p53 family signaling pathway involving iASPP after stroke.

Puma and Bax are target genes of the p53 family and ASPP family [16, 46-48]. Puma, initially discovered as a p53 target gene [49], was implicated in the regulation of apoptosis and neuronal survival [50, 51]. In our *in vivo* study, downregulation of iASPP aggravated cerebral ischemia/reperfusion injury by inducing apoptosis mediated via Puma and Bax. Hypothermia can reverse this injury by upregulation of iASPP signaling.

### Limitations

There are several limitations in this study. First, due to a lack of the sequence of the iASPP gene in rats, we did not detect the role of iASPP *in vitro* by RNA interference to iASPP. Second, iASPP has several isoforms with different roles in human. Hitherto, only one mRNA sequence of iASPP was found in mice, so we just investigated this iASPP sequence, which is similar with the long isoform of iASPP in human. Third, since there was no significance in the infarct volume, neurological function and cell death between MCAO groups without RNA interference and MCAO groups with RNA interference either under normothermia or hypothermia, we did not include the stroke groups plus hypothermia or normothermia treatment as controls to detect the expression of iASPP and its targets. Last but not least, previous studies have shown that upregulation of iASPP may be involved in the development and progression of several cancers [31, 52, 53]. Here, the oncogenic possibility of iASPP was neglected. We were only interested in studying the downregulation of iASPP and did not explore the effect of iASPP overexpression after stroke in mice.

In conclusion, our study suggests that iASPP plays an important role in the neuroprotective effects of therapeutic hypothermia through inhibition of apoptosis in experimental stroke. Therefore, iASPP might be an effective treatment target for patients with cerebral ischemia.
